# Association of sports practice aspects with health risk behaviors in adolescents: a systematic review and meta-analysis

**DOI:** 10.1590/1984-0462/2025/43/2024094

**Published:** 2024-12-20

**Authors:** Jhonatan Gritten Campos, Michael Silva, Rafael Vieira, Eliane Denise Araújo Bacil, Ana Beatriz Pacífico, Murilo Bastos, Wagner de Campos

**Affiliations:** a Universidade Federal do Paraná, Curitiba, PR, Brazil.; b Universidade Federal do Rio Grande, Rio Grande, RS, Brazil.; c Universidade Estadual do Centro-Oeste, Guarapuava, PR, Brazil.

**Keywords:** Sports, Health risk behaviors, Adolescent, Prática esportiva, Comportamentos de risco à saúde, Adolescentes

## Abstract

**Objective:**

To conduct a systematic review and meta-analysis to verify the association of aspects of sports practice with health risk behaviors in adolescents.

**Data source:**

A systematic search was conducted of electronic manuscripts from the United States National Library of Medicine (PubMed)/ Medical Literature Analysis and Retrieval System Online (MEDLINE), Web of Science, Science Direct, and Scientific Electronic Library Online (SciELO) published from January 2015 to December 2022. Studies examining the association between sport and health risk behaviors in adolescents aged 11 to 19 years were included. This study was registered in the International Prospective Register of Systematic Reviews (PROSPERO) under number CRD42023392053.

**Data synthesis:**

In total, 22 studies fulfilled the inclusion criteria. The association of sports practice with sedentary behavior showed odds ratio (OR) values ranging from 0,61 to 0,92, tobacco use from 0,35 to 0,73, illicit drugs from 0,40 to 0,91, and reduced inadequate sleep on weekdays of 0.57 (95% confidence interval — 95%CI 0.52–0.63) and weekends 0.79 (95%CI 0.69–0.89). In the meta-analysis, sports practice was significantly associated with alcohol consumption for boys (OR 1,36; CI95% 1,09–1,70), and was inversely associated with tobacco use for boys and girls (OR 0,59; CI95% 0,56–0,61).

**Conclusions:**

Adolescents who practice sports tend to have lower occurrences of sedentary behavior, tobacco and illicit drug use, and adequate amounts of sleep; and, in the meta-analysis, boys present higher values for alcohol consumption and boys and girls present lower values for tobacco use.

## RESUMO

**Objetivo:**

Realizar uma revisão sistemática e metanálise para verificar a associação de aspectos da prática esportiva com comportamentos de risco à saúde em adolescentes.

**Fontes de dados:**

Realizou-se uma busca sistemática de manuscritos eletrônicos da United States National Library of Medicine (PubMed)/ Medical Literature Analysis and Retrieval System Online (MEDLINE), Web of Science, Science Direct, and Scientific Electronic Library Online (SciELO), publicados de janeiro de 2015 a dezembro de 2022. Foram incluídos estudos que examinaram a associação entre o esporte e comportamentos de risco à saúde, com amostra composta de adolescentes com idades de 11 a 19 anos. Este estudo foi registrado na *International Prospective Register of Systematic Reviews* (PROSPERO) com número CRD42023392053.

**Síntese dos dados:**

Ao todo, 22 estudos preencheram os critérios de inclusão. A associação da prática esportiva com comportamento sedentário apresentou valores de razão de chances (*odds ratio* — OR) variando de 0,61 a 0,92, consumo de tabaco de 0,35 a 0,73, drogas ilícitas de 0,40 a 0,91 e redução do sono inadequado durante a semana de 0,57 (intervalo de confiança de 95% — IC95% 0,52–0,63) e finais de semana 0,79 (IC95% 0,69–0,89). Na metanálise, a prática esportiva teve associação significativa com o consumo de álcool para meninos (OR 1,36; IC95% 1,09–1,70) e esteve inversamente associada com o consumo de tabaco para meninos e meninas (OR 0,59; IC95% 0,56–0,61).

**Conclusões:**

Adolescentes que realizam prática esportiva tendem a apresentar menor ocorrência de comportamento sedentário, consumo de tabaco e de drogas ilícitas e quantidade de sono inadequada; e, na metanálise, os meninos apresentam valores mais elevados para o consumo de álcool e meninos e meninas valores mais baixos para o uso de tabaco.

## INTRODUCTION

Adolescence is a period of life in which several psychosocial and biological transformations occur in the individual, and this stage is the crucial time for establishing a healthy lifestyle.^1^ The behaviors acquired at this stage tend to perpetuate into adulthood. Thus, lifestyle decisions during adolescence are essential to choosing behaviors impacting an individual’s health and quality of life in the current and future years.^1^ Sedentary behavior (SB), alcohol consumption, smoking, illicit drugs use, and low quantity and quality of sleep are essential behaviors that can impact adolescents’ health and quality of life. On the other hand, the habitual practice of physical activity is linked to several health benefits in this age group.^2^

From the perspective of adolescent health promotion, through appropriate levels of physical activity, the practice of sports fits as an activity that meets the needs of this population, having positive effects mainly in the physical, physiological, and psychological domains. This activity is gaining more and more followers and, therefore, the emergence of young athletes is increasingly evident in sports centers.^3^

However, sports practice’s real benefits and consequences in the adolescent population are still controversial, especially when considering health risk behaviors (HRB) in individuals who practice regularly. The literature presents inconsistent results regarding some HRB. Some studies report that the practice of sports favors greater consumption of alcohol and tobacco^4-10^ and lower sleep quantity and quality.^11,12^ Other studies point to the practice of sports as a protective factor against the consumption of alcohol and tobacco use^1,11,13-15^ and as promoting better sleep quality.^16^ Yet some studies report no differences between groups of adolescents practicing and not practicing sports regarding sleep in general.^1,17^ For SB and illicit drugs, sports practice seems to favor a decrease in both HRB.^2,8,10,15,17,18^ Such differences in results may also be due to methodological discrepancies in the studies, even though they all involved adolescents, the sample size varied from 196^1^ to 60,601,^10^ and even though the majority were carried out in Europe,^4,6-8,11,14,15^ some studies were conducted in North America.^5,9,10,13^ Such factors may have led to the divergences in the results.

To date, no study has systematically investigated the association of sports practice with HRB in adolescents. In this sense, a broad search for scientific evidence that addresses the associations of sports for adolescent health is necessary since the literature is not consistent regarding whether sports practice is beneficial or not for this population. Therefore, this study aimed to conduct a systematic review and meta-analysis to verify the association of aspects of sports practice with HRB in adolescents.

## DATA SOURCE

The systematic review and meta-analysis were conducted according to the guidelines of the Preferred Reporting Items for Systematic Reviews and Meta-Analyses (PRISMA) protocol and registered in the International Prospective Register of Systematic Reviews (PROSPERO) under number CRD42023392053.

A systematic search of peer-reviewed manuscripts published from January 2015 to December 2022 was conducted. The Population, Intervention, Comparison, Outcomes (PICO) strategy was used to formulate the guiding question, with the following elements: population — adolescents; intervention — sports practice; comparison — practice or not practice; and outcomes — HRB. It was performed in the following databases: United States National Library of Medicine (PubMed), Scientific Electronic Library Online (SciELO), Science Direct and Web of Science.

Keywords and descriptors were used for the search process. Medical subject headings (MeSH) were used to determine descriptors for searching the title words, abstracts, and full articles. Keywords and descriptors were adjusted for each database using the Boolean operators AND OR.

The search syntax was: (sport) AND (“sedentary behavior” OR “screen time” OR “alcohol use” OR “alcohol consumption” OR “drinking behavior” OR “smoking” OR “tobacco use” OR “substance abuse” OR “drug use” OR “sleep” OR “sleep habits”) AND (“adolescents”).

This review evaluated studies that met the following inclusion criteria:

1. Manuscripts examining the association between sport (participation in sports activities, sports practice) and HRB (sedentary behavior, alcohol consumption, tobacco consumption, illicit drug consumption, and sleep quantity and quality);
2. Manuscripts published in Portuguese, English and Spanish;
3. Manuscripts using cross-sectional designs;
4. Manuscripts examining a sample of participants composed of adolescents aged 11 to 19 years.

Studies were excluded if they did not include children and adolescents within the proposed age range, studies involving children with special health conditions or who did not present association results.

After reviewing the databases, two authors independently screened the manuscripts based on the titles and abstracts of all identified records. Each author produced a list of manuscripts to examine the association between sports participation and HRB.

Any sports participation information was extracted that reported, for example, the volume of weekly practice, how long the adolescents had been practicing, whether they played individual or team sports, etc. For the HRB, the studies had to contain at least one variable from HRB: a variable of sports practice with sedentary behavior, consumption of alcohol, tobacco, or illicit drugs, or related to the quantity and quality of sleep.

The two lists were compared, consensus resolved discrepancies, and duplicates were removed. Articles that met the inclusion criteria were further evaluated during the following phase of the screening process. The same two authors independently read the full text of each manuscript to determine eligibility against the four inclusion criteria. Another consensus meeting occurred between the two authors to select manuscripts to be included in the final quantity and quantitative summaries. If consensus was not reached, a third reviewer was called in. The references of the final manuscripts were checked to identify articles that were not captured in the search.

Two authors independently extracted the following data: authors, year of publication, location, sample size (n), the instrument for sports participation and HRB, and main results.

The quality of the study and the reporting of each manuscript, along with the risk of bias, were assessed using the Appraisal Tool for Cross-Sectional Studies (AXIS).^19^ More specifically, this tool assesses the clarity of the study objectives, the adequacy of research methods, including study design, sample size and selection, measurement validity, and reliability, the adequacy of data analysis procedures, description of results, discussion of results, and possible conflicts of interest. Three reviewers independently evaluated each research manuscript. Ratings for each of the 20 AXIS items were compared during a meeting, and consensus resolved disagreements. Papers scoring close to 20 were considered of better quality.

A meta-analysis was performed to verify the evidence of association between aspects of sports practice with HRB in adolescents. The meta-analysis used the R software “meta” package version 3.5 and the RStudio interface. Odds ratio (OR), standard error (SE), and 95% confidence intervals (95%CI) were extracted and pooled using fixed and random effect models. The I^2^ test assessed heterogeneity among studies. I^2^ values of approximately 25, 50, and 75% indicate low, moderate, and high heterogeneity. Subgroup analyses were performed for sex and sport exposure.

## DATA SYNTHESIS

**Figure 1:**
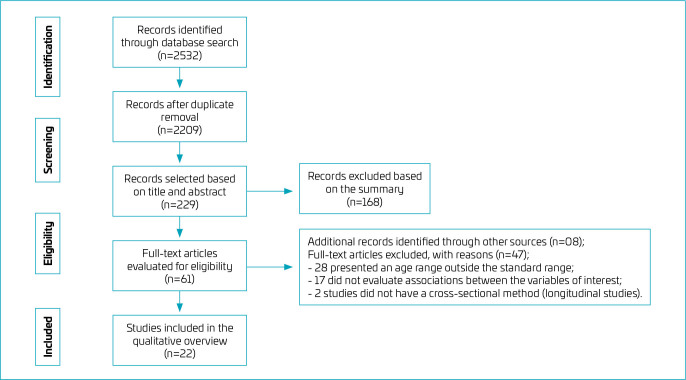
Flowchart of the manuscripts review process.

The initial search of manuscripts resulted in 2,532 research articles, and eight studies were added from analysis of article references, for a total of 2,540 articles. Duplicate analysis removed 323 studies. After screening the title and abstract, 53 articles were selected for full reading. Among the articles read, 23 were excluded because the age range was outside the chosen pattern, 14 did not present the associations between the variables of interest, and two did not present the cross-sectional design. Thus, 22 studies were included in the systematic review (Figure 1).

All manuscripts selected for final analysis ranged from January^2^ to November 2022.^12,20^ Of these studies, ten were conducted in North America,^3,5,9-13,20-22^ ten in Europe^1,4,6-8,14,15,17,23,24^ and two in South America.^18,25^ The age of the sample ranged from 11^4^ to 19 years.^8^ The sample size ranged from 196^11^ to 66,434^20^ thousand.

The selected manuscripts contained information on the association of at least one sport variable with one or more of the HRB. Among the sporting variables, the studies mentioned the weekly training volume (training hours and number of times a week),^1,11,13,15,17,18,21,23,24^ if the adolescents practice or have practiced sports in clubs,^1,3,5,9,10,14,20,22,25^ what type of sport they practice (individual and/or team sports)^4,7,18,23,24^ and how long they have practiced sports.^7,8,12,18^ Still, one study did not specify the questions addressed on this subject^6^. All these questions addressed in the studies could be applied to individual and/or team sports.

Regarding the HRB evaluated, four studies contained the variables of SB,^15,17,18,25^ ten consumption of alcoholic beverages,^3-8,10,18,21,23^ nine tobacco use,^3,4,8-10,15,20,22,24^ three consumption of illicit drugs^8,10,15^ and seven the duration and quality of sleep.^1,11-15,17^

Among the HRB variables, studies have cited screen time of two hours or more,^15,17,18^ sedentary time on a normal school day^18^ and sedentary time during school recess.^25^

For alcohol consumption, the most frequently mentioned questions ranged from recent consumption (past 30 days)^4,5,10,18,21^ to heavy drinking (four or five doses or more in the past 30 days on a single occasion).^3,18^ For tobacco use, it varied between having ever smoked in life,^3,15,20,22^ having done so recently (last 30 days)^3,4,9,10,20,22^ and doing so daily.^8,15,24^

Regarding the consumption of illicit drugs, the adolescents were asked whether or not they were users^8^ and if they had used them in the last 12 months.^10,15^ Regarding the quantity and quality of sleep, the questions most often asked were about the average amount^12,13,15,17^ and the quality of sleep.^1,11,12,14^

Regarding the instruments used to assess sports practice, most studies (17)^1,4,6-15,17,18,20,22,24^ did not present a validated instrument; rather, the authors selected specific questions to be asked, as mentioned above. In the remaining studies (5),^3,5,21,23,25^ the instruments selected were the Health Behaviour in School-aged Children (HBSC),^3^ the European Longitudinal Study of Pregnancy and Childhood (ELSPAC),^5^ PRIDE,^21^ Youth Risk Behavior Surveillance (YRBS)^25^ and International Physical Activity Questionnaire (IPAQ).^23^

For HRB, most studies used validated instruments. For SB, the most used instrument was the YRBS.^18,25^ For drinking, the most used instruments were the Alcohol Use Disorders Identification Test (AUDIT)^6-8^ and the HBSC.^1,4^ For tobacco consumption, the following i nstruments were used: HBSC^1,4^ and the Canadian Health Measures Survey (CHMS).^20,22^ For illicit drug use, the AUDIT^8^ and the European School Survey Project on Alcohol and Other Drugs (ESPAD)^15^ were used. For the amount of sleep, most studies asked only a question about bedtime and wake-up time^11-13^ or used an accelerometer to measure sleep duration.^1,17^ For sleep quality, the Pittsburgh Sleep Quality Index (PSQI)^1,11,14^ was the most used instrument.

The quality of studies was measured using the AXIS. On a scale of zero (low quality) to 20 points (high quality), the studies ranged from 11^24^ to 20 points^3^ (more detailed information is available upon request with the corresponding author), demonstrating the high quality of the studies analyzed in this systematic review and meta-analysis.

In Table 1 ^1,11-15,17,18,25^, the associations between aspects of sports practice with SB, quantity and quality of sleep are presented. Regarding SB, the association between higher weekly training volume and SB ranged from OR: 0.61 to 0.92.^15,17,18,25^ Regarding the amount of sleep, the evidence indicates that the practice of sports was positively associated with an adequate amount of sleep in the three studies presented.^11,13,15^ A study carried out with adolescents showed that sports practice was positively correlated with optimal sleep duration (r: 0.22).^11^ In another, a higher weekly frequency of organized sports practice was associated with less chance for low sleep duration on weekdays: OR 0.57 (95%CI 0.52–0.63) and less chance for low sleep duration on weekend days: OR 0.79 (95%CI 0.69–0.89).^15^ Moreover, this study showed a positive association between sports practice and hours of sleep on weekdays and weekends.^13^ And no significant association values were found in two studies.^1,17^ Furthermore, practicing sports was associated with good sleep quality in two studies^11,14^ (with a score of 4.1, where scores below 5 are classified as good quality and OR: 1.50 for adolescents who did not practice sports) and poor quality in one study^12^ (β=1.12).

**Table 1:** Studies that have investigated the association of sedentary behavior, quantity and quality of sleep with sports practice.

Reference	Country	Sample - n (age/grade level)	Sport participation	HRB evaluation	Main results	Axis score
Campos et al.^18^	Brazil	367 (average of 15.7 years)	Self-reported questionnaire	Sedentary behavior	Negative association	16
Saevarsson et al.^17^	Iceland	315 (average of 16 years)	Self-reported questionnaire	Sedentary behavior and quantity of sleep	Negative association and no significant association	17
Silva and Silva^25^	Brazil	2243 (13–18 years)	Self-reported questionnaire	Sedentary behavior	Negative association	14
Torstveit et al.^15^	Norway	13,269 (13–16 years)	Self-reported questionnaire	Sedentary behavior and quantity of sleep	Negative and positive association	15
Anderson and Reale^11^	USA	196 (average of 15.7 years)	Self-reported questionnaire	Quantity and quality of sleep	Positive association for both	12
Beltran-Valls et al.^1^	Spain	267 (average of 13.9 years)	Self-reported questionnaire	Quality of sleep	No significant association	16
von Rosen et al.^13^	USA	1016 (average of 17 years)	Self-reported questionnaire	Quantity of sleep	Positive association	15
Tekcan et al.^14^	Turkey	400 (14–18 years)	Self-reported questionnaire	Quality of sleep	Positive association	15
Watson et al.^12^	USA	1482 (14–18 years)	Self-reported questionnaire	Quality of sleep	Negative association	16

HRB: health risk behaviors; USA: United States of America. More detailed information is available upon request.

In Table 2 ^3-8,10,18,21,23^, for the consumption of alcoholic beverages, most studies^3,5-8,10^ indicate that sports practice favors consumption, with OR ranging from 1.21^3^ to 2.85^8^ for girls and from 1.33^3^ to 1.71^10^ for boys. However, other studies indicate that sports practice favors a decrease in this behavior, with OR ranging from 0.48 to 0.95.^4,18,21,23^

**Table 2:** Studies that have investigated the association of alcohol consumption with sports practice.

Reference	Country	Sample - n (age/grade level)	Sport participation	HRB evaluation	Main results	Axis score
Badura et al.^4^	Czech Republic	10,279 (11–15 years)	Self-reported questionnaire	Alcohol	Negative association	17
Boyes et al.^3^	Canada	13,817 (14–15 years)	Self-reported questionnaire	Alcohol	Positive association	20
Campos et al.^18^	Brazil	367 (average of 15.7 years)	Self-reported questionnaire	Alcohol	Negative association	16
Hryhorczuk et al.^5^	USA	1075 (average of 16.2 years)	Self-reported questionnaire	Alcohol	Positive association	18
King et al.^21^	USA	37,716 (13–18 years)	Self-reported questionnaire	Alcohol	Negative association	14
Peric et al.^6^	Croatia	267 (average of 16.3 years)	Self-reported questionnaire	Alcohol	Positive association	13
Sajber et al.^7^	Kosovo	1002 (17–18 years)	Self-reported questionnaire	Alcohol	Positive association	18
Tahiraj et al.^8^	Kosovo	980 (17–19 years)	Self-reported questionnaire	Alcohol	Positive association	14
López Villalba et al.^23^	Spain	564 (14–17 years)	Self-reported questionnaire	Alcohol	Negative association	15
Williams et al.^10^	Canada	60,601 (13–17 years)	Self-reported questionnaire	Alcohol	Positive association	15

HRB: health risk behaviors; USA: United States of America. More detailed information is available upon request.

In Table 3 ^3,4,8-10,15,20,22,24^, regarding tobacco and illicit drug use, most evidence suggests that sports practice was associated with a reduction in these behaviors, both for tobacco use (OR 0.35–0.73)^3,4,8-10,15,20,22,24^ and illicit drug use (OR 0.40–0.91).^8,10,15^

**Table 3:** Studies that have investigated the association of tobacco and illicit drug use with sports practice.

Reference	Country	Sample - n (age/grade level)	Sport participation	HRB evaluation	Main results	Axis score
Badura et al.^4^	Czech Republic	10,279 (11–15 years)	Self-reported questionnaire	Tobacco	Negative association	17
Boyes et al.^3^	Canada	13,817 (14–15 years)	Self-reported questionnaire	Tobacco	Negative association	20
De Nitto et al.^24^	Italy	327 (average of 16 years)	Self-reported questionnaire	Tobacco	Negative association	11
Irvine et al.^20^	Canada	66,434 (14–17 years)	Self-reported questionnaire	Tobacco	Negative association	18
Milicic et al.^22^	Canada	38,977 (14–18 years)	Self-reported questionnaire	Tobacco	Negative association	18
Tahiraj et al.^8^	Kosovo	980 (17–19 years)	Self-reported questionnaire	Tobacco and illicit drugs	Negative association for both	14
Torstveit et al.^15^	Norway	13,269 (13–16 years)	Self-reported questionnaire	Tobacco and illicit drugs	Negative association for both	15
Veliz et al.^9^	USA	4450 (12^th^ grade)	Self-reported questionnaire	Tobacco	Negative association	15
Williams et al.^10^	Canada	60,601 (13–17 years)	Self-reported questionnaire	Tobacco and illicit drugs	Negative association for both	15

HRB: health risk behaviors; USA: United States of America. More detailed information is available upon request.

**Figure 2:**
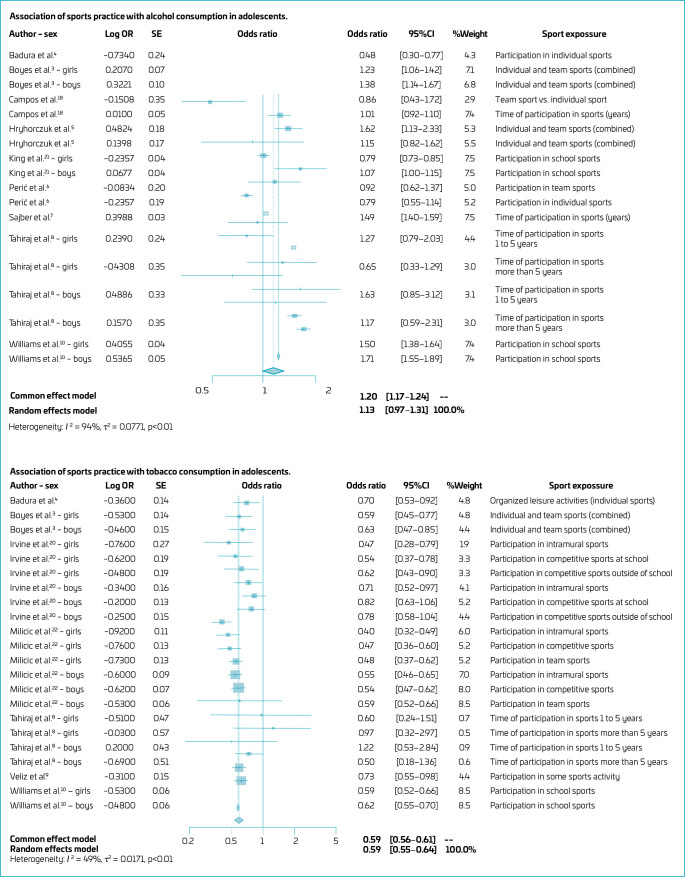
Association of sports practice with alcohol and tobacco consumption in adolescents.

Figure 2 presents the aggregate results of the associations between sports exposure variables and alcohol and tobacco consumption in adolescents. Exposure to sports did not show a significant association with alcohol consumption (OR 1.13; 95%CI 0.97–1.31 — random effect model). Given the high heterogeneity (I^2^=94%) found, subgroup analyses were performed so that exposure through alcohol consumption was found only for boys (OR 1.36; 95%CI 1.09–1.70) (Table 4). However, exposure to sports was inversely associated with tobacco use (OR 0.59; 95%CI 0.56–0.61).

**Table 4:** Subgroup analysis association of sports practice with alcohol consumption in adolescents.

	n	OR	95%CI	I^2^ (%)	Q	p-value
Subgroup						
Sex						
Boys	5	1.36*	**1.09–1.70**	93.2	59.0	<0.001
Girls	5	1.09*	0.81–1.45	96.8	125.7	<0.001
Sport exposure						
Sport participation	7	1.05*	0.79–1.38	76.9	26,0	0.002
School sports	4	1.21*	0.86–1.70	98.5	194.9	<0.001
Time practicing sports	6	1.20*	0.95–1.50	90.8	54.36	<0.001

n: number of articles; OR: odds ratio; 95%CI: confidence interval of 95%.

*Random effect model. Bold indicates statistically significant p-values.

## DISCUSSION

According to the reviewed studies, it can be identified that, within the sports variables, the weekly training volume decreases the likelihood of adolescents displaying SB. In a study with adolescents that play video games for long periods of time, negative associations were observed for weekly training volume (OR 0.92; 95%CI 0.86–0.99).^18^ Another study reported that the more often an adolescent practices sports (4–7x/week), the lower his odds of presenting SB in the form of screen time, compared to adolescents who practice sports three times weekly, thus demonstrating the importance of sports participation.^17^

Adolescents who do not participate in sports tend to have a 40% higher probability (OR 1.40; 95%CI 1.10–1.80) of being sedentary during school recess compared with adolescents who participate in two or more sports teams.^25^ Evidence shows that a higher weekly frequency of sports practice is associated with 39% lower involvement in SB.^15^

This transitional period from childhood to adolescence can be a delicate period for changes in behavior, such as exchanging more active leisure activities for more sedentary habits like watching television, playing video games, and using the computer. Besides increasing physical activity levels, the practice of sports tends to decrease screen time, thus being an essential tool for improving and maintaining adolescent health.^26^

The results of this study indicate that exposure to sports practice did not show a significant association with alcohol consumption; however, when analyzed separately by gender, it was possible to verify a significant association between exposure to sports and alcohol consumption in male adolescents (OR 1.36; 95%CI 1.09–1.70). In the literature, most studies^4-8,10^ indicate that adolescents who engage in sports are more likely to drink alcohol. On the other hand, some studies^3,18,21,23^ report that sports favor a decrease in this behavior.

The present study’s findings report significant associations for alcohol consumption only for male adolescents. The literature shows similar results.^3,6,10^ This may occur because such sports are identified with greater participation of boys, and it is more acceptable in society for boys to drink more than girls, and, in addition, boys gather more often in social groups for events than girls.^3,9^

Such results point in a direction where the relationship of sports practice to the use of alcoholic beverages may be linked to peer interaction and a drinking culture associated with many sports, since the consumption of alcohol is a socially acceptable form of celebration. Sports present many opportunities for this type of situation.^27^

An educational program aimed at athletes, coaches, and parents that highlights the negative effects of substance use on both athletic performance and health can be effective in preventing alcohol use. It is essential that public awareness campaigns, along with educational programs, are specifically developed for key groups involved in youth sports, such as parents, coaches, and sports organizations.^3,27^

Regarding tobacco consumption, sports practice is negatively associated with this behavior in all the studies presented,^3,4,8-10,15,20,22^ contributing to its non-occurrence. The meta-analysis shown in this study is in line with these findings, where exposure to sports practice was inversely associated with tobacco consumption (OR 0.59; 95%CI 0.56–0.61).

Such findings indicate that the practice of sports tends to decrease the occurrence of adolescent smoking; this may be because sports themselves are an effective tool against smoking. Some authors also point out that sports require a greater cardiorespiratory capacity; thus tobacco use can significantly affect performance during practice, as well as cause immediate physical disabilities (for example, shortness of breath), which would negatively affect health and, consequently, performance in sports.^9^

Sports help the physiological development and well-being of adolescents, and it can be expected that adolescents involved in sports practice are less likely to smoke and, consequently, to maintain a good performance in the sport they practice.^28^

Regarding the consumption of illicit drugs and tobacco consumption, sports practice favored the reduction of this behavior in all the studies presented. The literature highlights that adolescent participation in sporting activities is associated with lower use of illicit drugs, and playing sports was shown to be associated with lower odds (OR 0.40; 95%CI 0.30–0.52) of marijuana consumption.^15^

Individuals who do not use illicit drugs before the age of 21 have a lower propensity to use them later in life. Sports favor a lower occurrence of this behavior, thus the chances of consumption in adulthood are reduced.^8^

The literature also shows that adolescents who practice sports tend to identify themselves as athletes, and this may be a protective effect against the use of illicit drugs;^10,29^ therefore, intervening in the use of illicit substances through sports may represent a unique opportunity to reach a good portion of adolescents.^10^ Efforts during adolescence are essential to develop public health policies to prevent substance abuse in this population.

Sports practice was consistent for quantity^13,15,30^ but not for quality of sleep.^11,29^ Regarding the amount of sleep, a study^13^ showed that teenagers who played sports had an adequate duration of sleep, in addition to having a more significant amount of sleep compared to teenagers who did not play sports, who still did not meet the minimum recommended amount of sleep (8.5 vs. 7.7 hours/day).

In another study,^15^ the authors reported that those who practice sports have decreased odds for low sleep duration on weekdays (OR 0.57; 95%CI 0.52–0.63) and weekends (OR 0.79; 95%CI 0.69–0.89). This may be associated with a high need for sleep due to the energy demands of sports, which leaves practitioners more tired.

Adolescents must get a good amount of sleep because low sleep (<8 hours/day) during adolescence can lead to inattention, reduced executive functioning, increased risk of obesity and mood disorders, as well as occupational and sports injuries.^16^

Regarding sleep quality, the findings were not consistent in the literature. However, most studies demonstrate a favorable outcome.^11,14,31,32^ In a study with teenagers, 68% of those who played sports tended to have good quality sleep.^11^ These findings corroborate the results of other works^14,31,33,34^ by pointing out that adolescents who do not play sports are 1,5 times more likely (OR 1.50; 95%CI 1.00–2.23) to have poor sleep quality than those involved in sports,^14^ and athletes report better sleep patterns, including better sleep quality, shorter sleep onset latency and fewer awakenings after sleep onset, as well as less tiredness and greater concentration during the day compared to adolescents who do not participate in sports.^31^

In a study with adolescents, those involved in at least 150 minutes of physical activity or sports per week have a higher rate of good sleep quality,^32^ corroborating another study where 71.8% of adolescent athletes reported of good sleep quality.^33^ Sports practice is also associated with improved sleep quality by altering many factors, for example, shortening the latency to sleep onset.^34,35^ Such physical activities, even if not specifically related to sports, have positive effects on the quality of sleep in young people. Scientific evidence has shown an average PSQI score of 4.6 (indicating good sleep quality); however, 41% of the teenagers who played sports in this study reported sleeping poorly.^36^ This confirms other findings where high sports specialization was associated with greater daytime sleepiness.^12^

The present study’s findings should be analyzed with caution and are not free of limitations. The articles analyzed in this study used self-reported measures that depend on the individuals’ understanding of the evaluated variables. Although the instruments have adequate psychometric properties, they may increase the values. For this study, the inclusion criteria included only the analysis of observational studies with a cross-sectional characteristic. The remaining study designs were not considered in this systematic review and meta-analysis. New studies may involve other study designs, such as longitudinal and intervention studies, which are essential for determining the causality of the relationship between variables. Methodological differences in the analysis of sports variables were also observed among the studies. Furthermore, this work involved teenagers aged 11 to 19. The age difference causes them to present different behaviors, making it difficult to compare them. As strong points, we can highlight the performance of a systematic review and meta-analysis on the variables of risk behaviors and sports practice that, to this point, have not yet been found in the literature.

It is concluded that the practice of sports is favorable to a decrease in SB, tobacco consumption, and illicit drugs, as well as being beneficial for adequate sleep. Regarding the consumption of alcoholic beverages and quality of sleep, the results were inconsistent. From these results, it is evident that there is a need to create public policies to promote sport in childhood and adolescence to reduce HRB at this stage of an individual’s development. We emphasize the need for more studies that evaluate the association of sports practice with SB and sleep variables and analyze recreational and professional sports practice separately.

## Data Availability

The database that originated the article is available with a corresponding author.
